# Mental health, coping, and social support among people living with HIV in the Americas: A comparative study between Argentina and the USA during the SARS-CoV-2 pandemic

**DOI:** 10.21203/rs.3.rs-109131/v1

**Published:** 2020-11-18

**Authors:** Deborah L. Jones, Jamile Ballivian, Violeta J. Rodriguez, Claudia Uribe, Diego Cecchini, Ana S. Salazar, Isabel Cassetti, Maria L. Alcaide

## Abstract

**Background:** The COVID-19 pandemic pose significant risk to mental health and may disproportionately affect people living with HIV (PLWH). This study examined the interaction of social support and resilient coping in predicting depressive symptoms among PLWH. **Methods** : PLWH residing in Buenos Aires, Argentina and in Miami, Florida (US) were asked to complete an anonymous survey on the impact of COVID-19. Statistical analysis included ordinary least squares regression. **Results:** A total of 1,554 participants were included. Mean age was 47.30 years; 63.7 % were men. A test of three-way interaction of social support resilient coping study site indicated differences by site (b = -0.63.862, p = .043010, 95% CI [-1.24, -0.02.205, 1.52]). In Argentina, at higher social support and resilient coping, depressive symptoms were lowest. At lower social support and resilient coping, depressive symptoms were highest. **Discussion:** The impact of COVID-19 on mental health illustrates the need to develop innovative strategies to support resilience and to enhance coping with stress and adversity among PLWH.

## Introduction

Since its first description in December 2019, SARS-CoV-2, the virus responsible for the coronavirus disease 2019 (COVID-19), has spread rapidly around the world and has caused over 34 million infections and 1.5 million deaths [1, 2]. The SARS-CoV-2 (COVID-19) pandemic is a global public health crisis posing numerous increasing health and economic challenges. At the time of this writing, October 2020, the epicenter of the pandemic lies in the Americas. Across continents, the response to the epidemic has varied from country to country [1]. In Argentina, the first case was diagnosed in March 2020 [3], and the government declared an enforced COVID-19 lockdown on March 20, 2020 [4], when there were 158 confirmed cases [5]. The lockdown extended over four months and resulted in slowing the progression of the epidemic in the country, with 903,730 total cases on October 12, 2020 [1, 3]. Conversely, the USA by March 2020 had already faced an exponential increase in laboratory-confirmed cases of COVID-19 and related deaths [6]. This surge led to many states and localities to issue stay-at-home orders, including orders for many nonessential businesses to close [7]. Today, after months of the initial outbreaks the epidemic continues to advance in both countries, placing them among the most affected nations by the SARS-CoV-2 pandemic in the Americas [1].

The first descriptions of the disease established that the infection could cause severe respiratory distress and illness [8, 9]. High-risk population for severe forms included people 60 years and older and those of any age with underlying medical conditions (e.g., chronic lung disease, asthma, heart conditions, etc) [10-12]. As such, people living with HIV infection (PLWH) are considered to be at greater risk for adverse outcomes due to COVID-19 [13-15]. Although recent retrospective analysis of hospitalized PLWH with

COVID-19 do not appear to show higher rates of infection or severity among PLWH, the literature is scarce and lacks longitudinal follow up [16-18].

The SARS-CoV-2 pandemic represents an unprecedented public health threat, and mitigation strategies such as enforced lockdown and physical distancing combined with anxiety about potential infection, recently dubbed ‘coronaphobia,’ can have a profound impact on mental health [19]. In Argentina, adherence to restrictions has decreased lately, primarily due to its significant financial impact in a country with a fragile economy where more than 35% of the population are living under the poverty line [20]. In the US, the economy has suffered its most severe contraction since the Great Depression with more than 26 million people filed for unemployment and in March 2020. As a result, the US government approved a $2.2 trillion economic stimulus bill to reactivate the economy. However, uncertainty regarding the eventual impact of the pandemic remains.

The long-term consequences of lockdown and economic disruption on mental health among PLWH around the world are unknown. Mental health, social support, and substance use are predictors of HIV-related health outcomes, including treatment adherence [21], and having a psychiatric diagnosis has been associated with increased mortality among patients hospitalized with COVID-19 [22]. National surveys have described the mental health of the population during pandemics [23, 24], including the COVID-19 pandemic [25]. However, there is limited information on the use of strategies that could mitigate the impact of the pandemic on PLWH such as resilience coping, social support, and there have been no comparisons between nations affected by the pandemic in the Americas. Thus, this study examines the experiences of PLWH living in Argentina and the USA during the SARS-CoV-2 pandemic and explores the role of social support and resilient coping on mitigating the pandemic’s mental health impact.

## Methods

Data collection for this cross-sectional study of PLWH in Argentina and the US was conducted during the first six months of mitigation of the SARS-CoV-2 pandemic (April – May 2020 in Argentina and April – September 2020 in the US). Following approval from the Institutional Review Boards (IRB) at the study sites, candidates were contacted regarding the study, provided with information on the study, and consented, before being administered or completing an adapted version of the MACS/WIHS Combined Cohort Study (MWCCS) [26] COVID-19 survey in English or Spanish.  At the Argentinian site, the questionnaire was also adapted to Argentine Spanish prior to administration. Individual study site procedures are described below.

#### Participants

Data from adults (>18 years) PLWH receiving HIV care at the participating sites, were collected from collaborative studies located in Buenos Aires (Argentina) and Miami (USA).

**Argentina:** PLWH candidates were identified using the *Helios Salud* database, derived from information obtained as nongovernmental providers of HIV outpatient healthcare services for more than 10,000 PLWH

residing mostly in Buenos Aires Metropolitan Area. Study staff contacted potential candidates via WhatsApp or email and notified candidates they could decline participation with no interference to their clinical services. The survey was self-administered and accessed through a web link using the REDCap© (Research Electronic Data Capture)[27] data capture platform.

**USA:** PLWH candidates were identified through the Miami Center for HIV Research in Mental Health (CHARM)/Center for AIDS Research (CFAR) patient database and enrolled in the ACTION study (A Comprehensive Translational Initiative on Novel Coronavirus Cohort) which was approved by the University of Miami (UM) IRB prior to study start. Participants resided in Miami, Florida, one of the cities with higher number of HIV and SARS-CoV-2 infections in the US [28]. The survey was administered by phone by study personnel or self-administered via web link if participants preferred

**Variables / Measures:** The COVID Survey was developed by the MWCCS team to address the experience of individuals living with, or at risk for, HIV infection. The survey included sociodemographics (age, gender, living arrangements), COVID-19 preventive behaviors and disruptions, mental health and psychosocial factors, including depression, perceived stress, loneliness, resilient coping, and social support [29-33].

**Psychosocial factors:** Depression symptomatology measurements were retrieved from the questions, "I felt lonely" and "I felt depressed", using responses on a 4-point Likert-type ordinal scale from 1 = Never to 4 = Always [29].

Perceived Stress was assessed using an adapted measure of the Perceived Stress Scale [30] and by the items: the "Difficulties were piling up so high that I could not overcome them?" and, "…unable to control the important things in life?". These items are rated on a 5-point Likert-type scale 1 = Never to 5 = Very often.

Loneliness [29] was measured using a 3-point Likert-type scale from 1 = Hardly Ever to 3 = Often. Items included, "How often do you feel that you lack companionship?", "How often do you feel isolated from others?", and "How often do you feel left out?". The sum of three items was used to calculate this score.

Resilient Coping was measured by an adaptation of the Brief Resilient Coping Scale [34]. Items included "I look for creative ways to alter difficult situations", "regardless of what happens to me, I believe I can control my reaction to it", and "I believe I can grow in positive ways by dealing with difficult situations". A 5-point Likert scale was used for item responses (1 = Does not describe me at all to 5 = Describes me very well). The sum of four items was used to calculate this score.

Social support [33]was measured using a 5-point Likert scale ranging from 1 = Very Dissatisfied to 5 = Very Satisfied ("In the last month, how satisfied are you with the social support that you have received from others?").

**Analytic Plan**

Descriptive analyses included mean and standard deviation for key study variables. Comparisons by Argentina and USA were performed using t-tests, chi-square tests, or non-parametric alternatives. A three-way interaction of social support   resilient coping   study site predicting depressive symptoms was tested using the Model 2 PROCESS macro by Hayes for SPSS [35]. To prevent multicollinearity between variables, mean centering was performed on all variables. To establish statistical significance, a threshold cutoff value of $p < .05$ was applied. All statistical analyses were conducted using Statistical Package for the Social Sciences version 26.

## Results

#### *Demographic, COVID-19, Depression, and Psychosocial Variables*

A total of 1,554 participants were included in the study, n=1,336 from Argentina and n=218 from the US. On average, participants were 47.30 (SD = 10.68) years old, and USA participants were older (*M* = 53.32; *SD* =10.88) compared to participants from Argentina (*M* = 46.32; *SD* = 10.32). Two-thirds of participants were men, with a greater proportion of men in Argentina (67% versus 45%, $X^2$ (1) = 38.57, < .001). The majority of participants lived in their own home or apartment (82%), with a greater proportion in Argentina (83%) than the USA (76%).

With regards to COVID-19 preventive behaviors and disruption, there was high social distancing compliance reported but a greater proportion of participants reported practicing social distancing in Argentina (99%) than in the USA (95%) (p<0.001). Rates of participants who quarantined due to having symptoms or an exposure were low. A greater proportion of participants in the USA reported being quarantined due to COVID-19 symptoms (13% versus 2%, respectively), contact with someone with COVID-19 (12% versus 1%), or due uncertainty about whether they were positive for COVID-19 (12% versus 4%). There was a greater proportion of participants who had lost a job or worked less hours due to COVID-19 in Argentina (62%) than in the USA (42%), but a greater proportion of participants in the USA had difficulty paying for basic needs (66%) than in Argentina (59%).

Participants in the USA reported higher levels of depressive symptoms (*M* = 13.38 (*SD* = 5.98) versus *M* = 12.78 (*SD* = 4.26)), but higher levels of resilient coping (*M* = 11.88 (*SD* = 2.32) versus *M* = 11.32 (*SD* = 2.88)) (p<0.05 for depressive symptoms and resilient coping). Details on demographic, COVID-19, mental health, and psychosocial variables is described in Table 1.

#### *Depressive Symptoms, Social Support, and Resilient Coping*

A test of three-way interaction of social support   resilient coping   study site was shown to be significant, (*b* = -0.63, *p* = .043, 95% CI [-1.24, -0.02]). In Argentina, but not in the US, at high levels of social support and resilient coping, depressive symptoms were the lowest. At low levels of social support and resilient coping, depressive symptoms were the highest. The three-way interaction is plotted in Figure 1. Full details of the model are in Table 2.

## Discussion

This study evaluated mental health and social support factors among PLWH in Argentina and the USA during the SARS-CoV-2 pandemic, and examined the interaction among depression, social support, and resilient coping. Results reveal that PLWH in the USA had higher levels of depression and resilient coping than those in Argentina. In Argentina, not in the US, social support and resilient coping contributed to lower levels of depression.

Directives to reduce the spread of SARS-CoV-2, such as national lockdowns and social distancing, pose significant wide-ranging effects on population mental health – particularly among vulnerable populations who may be more likely to comply. Previous studies have found that pandemics, including COVID-19, are associated with an increased risk of developing stress, depression, anxiety and loneliness [19, 36, 37], with up to 31% of people reporting at least one episode of depression related to the COVID-19 pandemic [23]. In addition, having mental health diagnosis is more common among PLWH [19], and has been associated with increased mortality among hospitalized patients with COVID-19 [22]. This study found that PLWH have high levels of depression, loneliness, and low levels of social support, which may negatively impact their overall health and HIV outcomes. Thus, this could shed light on the effect of the regional pandemic among the vulnerable population of PLWH. However, the longer-term consequences on mental health are largely unknown and will need to be evaluated among PLWH and vulnerable populations.

Social support, an important predictor of resilient coping, may play an important role in buffering the impact of major life challenges on emotional distress and behavior, especially during the SARS-CoV-2 pandemic. It has been previously reported that among PLWH in Argentina, resilient copying strategies mitigated emotional distress and was hypothesized that strategies to developed resilience skills could facilitate coping with the effect of the coronavirus pandemic in this population [38]. However, this effect was not identified among the PLWH in the US population in this study. A better understanding of how coping and social support relate to depression among PLWH in different regions may help to facilitate the implementation of regionally specific appropriate mental health treatment and support services.

The financial impact of the pandemic can be a risk factor for psychological disorders and poor health outcomes.[39, 40] Yet, Argentine PLWH in this study were able to cope and develop resilience in spite of widespread financial stress. It is conceivable that the severe economic crisis in Argentina in recent years has stimulated a greater adaptive response to job insecurity and financial hardship [41], or that financial assistance from the government during the pandemic has alleviated the stress due to financial burden [42]. In contrast, while PLWH in the US received financial assistance, many had higher levels of stress and depression. A previous study of people quarantined due to Ebola in Senegal found that, although participants received financial aid, some felt that the amount was inadequate, or it arrived too late; others felt that the help they received did not cover their continuing expenses [43]. As such, vulnerable individuals in the USA who were quarantined and had low household income or income loss may need other support to manage pandemic-related stress and depression in addition to financial assistance.

As hypothesized, social support was negatively associated with depressive symptoms, and this effect was strengthened by resilient coping, which differed by region. In Argentina, depressive symptoms were the lowest when having high levels of social support and resilient coping, whereas in the US, though the association between depressive symptoms and social support was present, it was not strengthened by resilient coping. Cultural differences might have influenced the perception of stress and the benefit of resilience coping skills in each region. Social support and adaptive coping that be regarded differently in different cultural contexts [40]. Previous studies have found that Hispanics tend to have better resilient coping mechanisms than non-Hispanic due to a trajectory of overcoming stress through character traits, cultural values, and larger social support [44]. Exploring cultural factors that promote resilience may enable a better understanding of the coping process, and with it, comprehensive interventions that maximize protective factors among the most vulnerable populations could be developed.

The primary limitations of this study include its cross-sectional design, which prevents causal inference. In addition, surveys were both online and self-reported and there is the potential of respondent bias. Finally, participants were primarily drawn from two regions in each country. As the severity of the pandemic and the availability of medical services varies in each country, these findings may not represent the impact of the pandemic in other settings. Future research is needed to address the longitudinal effect of resilience and long-term outcomes.

This early study in the USA and Argentina suggests that resilient coping may act as a protective factor against the array of challenges and adversities associated with SARS-CoV-2 among PLWH. Previous studies have shown that resilience needs to be fostered early to prevent the development of depressive symptoms.[40] As PLWH are a vulnerable population already at higher risk of mental health burden and of severe COVID-19, understanding the drivers of COVID-19 infection in this population is pivotal for developing timely and effective public health interventions and mitigate risks.

## Conclusion

The psychological impact of a pandemic for PLWH can be extensive and long lasting. Understanding the aspects of support that promote positive coping among vulnerable individuals living with HIV is important to supporting mental health and optimizing health outcomes. Initiatives are needed now to promote health and to empower PLWH with strategies to cope with stress and adversity.

## Declarations

#### Acknowledgement

**Funding:** This work was supported by National Institutes of Health grants to the University of Miami Center for AIDS Research grant (P30A1073961), the Center for HIV and Research in Mental Health (P30MH116867) and Helios Salud. VJR’s work on this manuscript was partially supported by a Ford Foundation Fellowship, administered by the National Academies of Sciences, Engineering, and Medicine.

**Conflicts of interest:** MLA is currently receiving a grant (#P30AI073961) from the Miami Center for AIDS Research (CFAR). DLJ is currently recieiving a grant (#P30MH116867) from the Clinic HIV/AIDS Registry of Miami (CHARMS). VJR was supported by a Ford Foundation Fellowship. For the remaining authors no competing interests were declared.

**Consent:** Informed consent was obtained from all individuals participants included in the study.

**Ethics approval:** The questionnaire and methodology for this study was approved by the Institutional Review Boards of the University of Miami and Helios Salud. This study was performed in accordance with the ethical standards as laid down in the 1964 Declaration of Helsinki.

**Data availability:** The data underlying this article will be shared on reasonable request to the corresponding author.

**Author's Contribution:** All authors had full access to all the data in the study and take responsibility for the accuracy and the integrity of the data analysis. MLA and DJ were responsible for study concept and design. All authors were responsible for data acquisition and interpretation of results. CU and VJR contributed with the data analyses. All authors were responsible for drafting and critical revision of the manuscript for important intellectual content.

## References

1. Johns Hopkins University Center for Systems Science and Engineering. Coronavirus COVID-19 Global Cases 2020 [Accessed: July 02, 2020] Available from: https://coronavirus.jhu.edu/us-map.
2. Centers for Disease Control and Prevention (CDC). United States COVID-19 Cases and Deaths by State: CDC. Coronavirus Disease 2019 (COVID-19). Covid Data Tracker. ; 2020 [Accessed: July 29 2020.] Available from: https://covid.cdc.gov/covid-data-tracker.
3. Ministerio de Salud de Argentina. Nuevo Coronavirus COVID-19 Argentina 2020 [Accessed: June 17, 2020] Available from: https://www.argentina.gob.ar/salud/coronavirus-COVID-19.
4. Kirby T. South America prepares for the impact of COVID-19. The Lancet Respiratory Medicine. 2020;8(6):551-2.
5. Statista. Rate of coronavirus (COVID-19) cases in Argentina 2020 [Accessed: July 22, 2020] Available from: https://www.statista.com/study/23060/argentina-statista-dossier/.
6. Dong E, Du H, Gardner L. An interactive web-based dashboard to track COVID-19 in real time. Lancet Infect Dis. 2020;20(5):533-4.
7. Fitch Solutions. Argentina In Covid-19 Lockdown: Impact On Consumer Sector. 220 [Accessed: April 09, 2020] Available from: https://www.fitchsolutions.com/corporates/retail-consumer/argentina-covid-19-lockdown.
8. Zhu N, Zhang D, Wang W, Li X, Yang B, Song J, et al. A Novel Coronavirus from Patients with Pneumonia in China, 2019. N Engl J Med. 2020;382(8):727-33.

9. Wu Z, McGoogan JM. Characteristics of and Important Lessons From the Coronavirus Disease 2019 (COVID-19) Outbreak in China: Summary of a Report of 72314 Cases From the Chinese Center for Disease Control and Prevention. JAMA. 2020;323(13):1239-42.
10. Dhama K, Patel SK, Pathak M, Yatoo MI, Tiwari R, Malik YS, et al. An update on SARS-CoV-2/COVID-19 with particular reference to its clinical pathology, pathogenesis, immunopathology and mitigation strategies. Travel Med Infect Dis. 2020:101755.
11. Onder G, Rezza G, Brusaferro S. Case-Fatality Rate and Characteristics of Patients Dying in Relation to COVID-19 in Italy. JAMA. 2020;323(18):1775-6.
12. Zheng Z, Peng F, Xu B, Zhao J, Liu H, Peng J, et al. Risk factors of critical & mortal COVID-19 cases: A systematic literature review and meta-analysis. J Infect. 2020.
13. Meyerowitz EA, Kim AY, Ard KL, Basgoz N, Chu JT, Hurtado RM, et al. Disproportionate burden of coronavirus disease 2019 among racial minorities and those in congregate settings among a large cohort of people with HIV. Aids. 2020;34(12):1781-7.
14. Blanco JL, Ambrosioni J, Garcia F, Martinez E, Soriano A, Mallolas J, et al. COVID-19 in patients with HIV: clinical case series. Lancet HIV. 2020;7(5):e314-e6.
15. Gervasoni C, Meraviglia P, Riva A, Giacomelli A, Oreni L, Minisci D, et al. Clinical features and outcomes of HIV patients with coronavirus disease 2019. Clin Infect Dis. 2020.
16. Benkovic S, Kim M, Sin E. Four cases: Human immunodeficiency virus and novel coronavirus 2019 Co-infection in patients from Long Island, New York. J Med Virol. 2020.
17. Ridgway JP, Farley B, Benoit JL, Frohne C, Hazra A, Pettit N, et al. A Case Series of Five People Living with HIV Hospitalized with COVID-19 in Chicago, Illinois. AIDS Patient Care STDS. 2020;34(8):331-5.
18. Calza L, Bon I, Borderi M, Colangeli V, Viale P. No Significant Effect of COVID-19 on Immunological and Virological Parameters in Patients With HIV-1 Infection. Journal of acquired immune deficiency syndromes (1999). 2020;85(1):e6-e8.
19. Shiau S, Krause KD, Valera P, Swaminathan S, Halkitis PN. The Burden of COVID-19 in People Living with HIV: A Syndemic Perspective. AIDS and behavior. 2020;24(8):2244-9.
20. Instituto Nacional de Estadistica y Censos de Argentina (INDEC). Incidencia de la pobreza y la indigencia en 31 aglomerados urbanos. Primer semestre de 2020 ISSN 2545-6660 Buenos Aires, Argentina: Ministerio de Economia; 2020 [Accessed: September 29, 2020] Available from: https://www.indec.gob.ar/informesdeprensa/eph_pobreza.
21. McMahon JM, Braksmajer A, Zhang C, Leblanc N, Chen M, Aidala A, et al. Syndemic factors associated with adherence to antiretroviral therapy among HIV-positive adult heterosexual men. AIDS Res Ther. 2019;16(1):32-.
22. Li L, Li F, Fortunati F, Krystal JH. Association of a Prior Psychiatric Diagnosis With Mortality Among Hospitalized Patients With Coronavirus Disease 2019 (COVID-19) Infection. JAMA Network Open. 2020;3(9):e2023282-e.
23. Czeisler MÉ LR, Petrosky E, et al.,. Mental Health, Substance Use, and Suicidal Ideation During the COVID-19 Pandemic— United States, June 24–30, 2020. Morbidity and Mortality Weekly Report

(MMWR),. 2020;69(69):1049-57.

24. Direccion Nacional de Salud Mental y Addiciones. Salud Mental COVID-19 Buenos Aires, Argentina2020 [Accessed: October 5, 2020] Available from: https://saludmentalcovid19.org/.
25. Galea S, Merchant RM, Lurie N. The Mental Health Consequences of COVID-19 and Physical Distancing: The Need for Prevention and Early Intervention. JAMA Internal Medicine. 2020;180(6):817-8.
26. Multicenter AIDS Cohort Study (MACS) and the Women's Interagency HIV Study (WIHS). MACS/WIHS Combined Cohort Study (MWCCS), 2019 [Accessed: April 23, 2020] Available from: https://mwccs.org/.
27. Harris PA, Taylor R, Thielke R, Payne J, Gonzalez N, Conde JG. Research electronic data capture (REDCap)–a metadata-driven methodology and workflow process for providing translational research informatics support. J Biomed Inform. 2009;42(2):377-81.
28. Florida Department of Health. HIV/AIDS Florida: Florida Health in Miami-Dade.; 2019 [Accessed: June 30, 2020] Available from: http://miamidade.floridahealth.gov/programs-and-services/infectious-disease-services/hiv-aids-services/.
29. Zhang W, O'Brien N, Forrest JI, Salters KA, Patterson TL, Montaner JSG, et al. Validating a Shortened Depression Scale (10 Item CES-D) among HIV-Positive People in British Columbia, Canada. PLoS ONE. 2012;7(7):e40793.
30. Cohen S, Kamarck, T., & Mermelstein, R. A Global Measure of Perceived Stress. Journal of Health and Social Behavior. 1983;24(4):385.
31. Russell D, Peplau LA, Cutrona CE. The revised UCLA Loneliness Scale: Concurrent and discriminant validity evidence. Journal of Personality and Social Psychology. 1980;39(3):472-80.
32. Cella D, Choi SW, Condon DM, Schalet B, Hays RD, Rothrock NE, et al. PROMIS((R)) Adult Health Profiles: Efficient Short-Form Measures of Seven Health Domains. Value Health. 2019;22(5):537-44.
33. Friedman MR, Coulter RW, Silvestre AJ, Stall R, Teplin L, Shoptaw S, et al. Someone to count on: social support as an effect modifier of viral load suppression in a prospective cohort study. AIDS Care. 2017;29(4):469-80.
34. Sinclair VG, Wallston KA. The Development and Psychometric Evaluation of the Brief Resilient Coping Scale. Assessment. 2004;11(1):94-101.
35. Hayes A. The PROCESS macro for SPSS and SAS. 2015.
36. Zhao H, He X, Fan G, Li L, Huang Q, Qiu Q, et al. COVID-19 infection outbreak increases anxiety level of general public in China: involved mechanisms and influencing factors. Journal of Affective Disorders. 2020;276:446-52.
37. Peng M, Mo B, Liu Y, Xu M, Song X, Liu L, et al. Prevalence, risk factors and clinical correlates of depression in quarantined population during the COVID-19 outbreak. J Affect Disord. 2020;275:119-24.

38. Ballivian J, Alcaide ML, Cecchini D, Jones DL, Abbamonte JM, Cassetti I. Impact of COVID-19-related stress and lockdown on mental health among people living with HIV in Argentina. JAIDS Journal of Acquired Immune Deficiency Syndromes. 9000;Publish Ahead of Print.
39. Brooks SK, Webster RK, Smith LE, Woodland L, Wessely S, Greenberg N, et al. The psychological impact of quarantine and how to reduce it: rapid review of the evidence. Lancet. 2020;395(10227):912-20.
40. Clauss-Ehlers CS. Sociocultural factors, resilience, and coping: Support for a culturally sensitive measure of resilience. Journal of Applied Developmental Psychology. 2008;29(3):197-212.
41. Administración Nacional de la Seguridad Social. Ingreso Familiar de Emergencia (IFT). Argentina Argentina: Presidencia Argentina; 2020 [Accessed: Oct 1, 2020] Available from: https://www.anses.gob.ar/ingreso-familiar-de-emergencia.
42. Ministerio de Salud de Argentina. COVID-19 - Asistencia de Emergencia al Trabajo y la Producción (ATP) Argentina2020 [Accessed: June 17, 2020] Available from: https://www.argentina.gob.ar/salud/coronavirus-COVID-19.
43. Pellecchia U, Crestani R, Decroo T, Van den Bergh R, Al-Kourdi Y. Social Consequences of Ebola Containment Measures in Liberia. PLoS One. 2015;10(12):e0143036.
44. Farley T, Galves A, Dickinson LM, Perez Mde J. Stress, coping, and health: a comparison of Mexican immigrants, Mexican-Americans, and non-Hispanic whites. J Immigr Health. 2005;7(3):213-20.

## Table

**Table 1. Demographic, COVID-19, Mental Health and Psychosocial Variables**

| | All $N = 1554$ | Argentina $n = 1336$ | USA $n = 218$ | $t/\chi^2$, $p$ |
|---|---|---|---|---|
| **Demographic Characteristics** | | | | |
| Age | 47.30 (10.68) | 46.32 (10.32) | 53.32 (10.88) | 2.62, .009 |
| Sex | | | | |
| Female | 564 (36.3%) | 444 (33.2%) | 120 (55.0%) | |
| Male | 990 (63.7%) | 892 (66.8%) | 98 (45.0%) | 38.57, < .001 |
| Living Arrangements | | | | |
| Own house/ apartment | 1268 (81.6%) | 1102 (82.5%) | 166 (76.1%) | |
| Other | 286 (18.4%) | 234 (17.5%) | 52 (23.9%) | 5.01, .025 |
| **COVID-19 Preventive measures and disruptions** | | | | |
| Staying at home | | | | |
| No | 52 (3.3%) | 43 (3.2%) | 9 (4.1%) | |
| Yes | 1502 (96.7%) | 1293 (96.8%) | 209 (95.9%) | 0.48, .489 |
| Practice social distancing | | | | |
| No | 22 (1.4%) | 12 (0.9%) | 10 (4.6%) | |
| Yes | 1532 (98.6%) | 1324 (99.1%) | 208 (95.4%) | 18.27, < .001 |
| Quarantined due to COVID-19 symptoms | | | | |
| No | 1505 (96.8%) | 1316 (98.5%) | 189 (86.7%) | |
| Yes | 49 (3.2%) | 20 (1.5%) | 29 (13.3%) | 85.54, < .001 |
| Quarantined due to contact with COVID-19 | | | | |
| No | 1522 (97.9%) | 1329 (99.5%) | 193 (88.5%) | |
| Yes | 32 (2.1%) | 7 (0.5%) | 25 (11.5%) | 111.30, < .001 |
| Quarantined due to uncertainty of COVID-19 infection | | | | |
| No | 1479 (95.2%) | 1288 (96.4%) | 191 (87.6%) | |
| Yes | 75 (4.8%) | 48 (3.6%) | 27 (12.4%) | 31.54, < .001 |
| Self or others in household lost job or worked less hours due to COVID-19 | | | | |
| No | 641 (41.2%) | 515 (38.5%) | 126 (57.8%) | |
| Yes | 913 (58.8%) | 821 (61.5%) | 82 (42.2%) | 28.66, < .001 |
| Difficulty paying basic needs due to COVID-19 | | | | |
| No | 624 (40.2%) | 550 (41.2%) | 74 (33.9%) | |
| Yes | 930 (59.8%) | 786 (58.8%) | 144 (66.1%) | 4.07, .044 |
| **Mental Health and Psychosocial Variables** | | | | |
| Depression | 12.84 (4.56) | 12.78 (4.26) | 13.38 (5.98) | 3.31, .001 |
| Stress | 8.27 (1.16) | 8.28 (1.11) | 8.19 (1.40) | 0.84, .405 |
| Loneliness | 3.74 (3.77) | 3.59 (1.17) | 4.86 (1.91) | 1.74, .082 |
| Resilient Coping | 11.40 (2.81) | 11.32 (2.88) | 11.88 (2.32) | 2.70, .007 |
| Social support | 4.02 (0.91) | 4.00 (0.89) | 4.16 (0.99) | 1.75, .080 |

## Figures

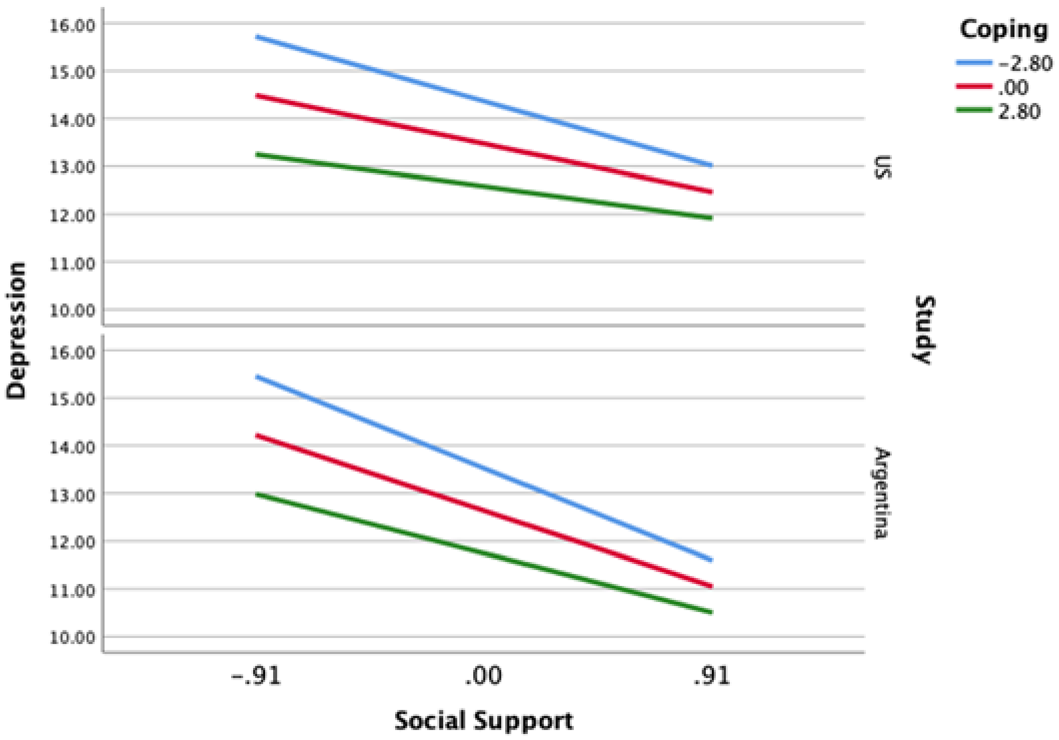

### Figure 1

Interaction of social support, resilient coping, and study site predicting depressive symptoms during the SARS-CoV-2 pandemic.
